# CMV in Hematopoietic Stem Cell Transplantation

**DOI:** 10.4084/MJHID.2016.031

**Published:** 2016-06-20

**Authors:** Rafael de la Cámara

**Affiliations:** Hospital de la Princesa C/Diego de León n° 62, Madrid 28006. Spain

## Abstract

Due to its negative impact on the outcome of stem cell transplant (SCT) and solid organ transplant patients (SOT) CMV has been called “the troll of transplantation”. One of the greatest advances in the management of SCT has been the introduction of the preemptive strategy. Since its introduction, the incidence of the viremia, as expected, remains unchanged but there has been a marked decline in the incidence of early CMV disease. However, in spite of the advances in prevention of CMV disease, CMV is still today an important cause of morbidity and mortality. Late CMV disease is still occurring in a significant proportion of patients and the so-called indirect effects of CMV are causing significant morbidity and mortality. Fortunately there have been several advances in the development of new antivirals, adoptive immunotherapy and DNA-CMV vaccines that might transform the management of CMV in the near future.

## Introduction

Today it is widely known that CMV is a very important pathogen in the transplant setting, but, curiously, it has not always has been considered this way. It is surprising to know that the first article that identified CMV as a major pathogen in transplant patients^1^ was rejected when it was first submitted for publication; the author was told that it was common knowledge that CMV does not cause disease.^2^ Unfortunately, we learned that this is not true, and CMV disease was for a long time the first cause of transplant-related mortality. Due to its negative impact on the clinical outcome of SCT and SOT it has been called “the troll of transplantation” by Prof Balfour in a very graphic description:^3^ “Cytomegalovirus is the troll under the bridge, hidden in shadows and often undetectable even by the most sophisticated diagnostic techniques. As we immunosuppress patients to help them cross the bridge, the troll comes out and threatens to devour them”. Now the incidence of CMV disease is pretty low (5%), so It could be logical to think that, today, CMV is not a big problem. As we will see, unfortunately this is not the case and CMV is still today an important cause of morbidity and mortality.

### a) Past and Present Situation

#### a1) CMV disease

**Figure 1:**
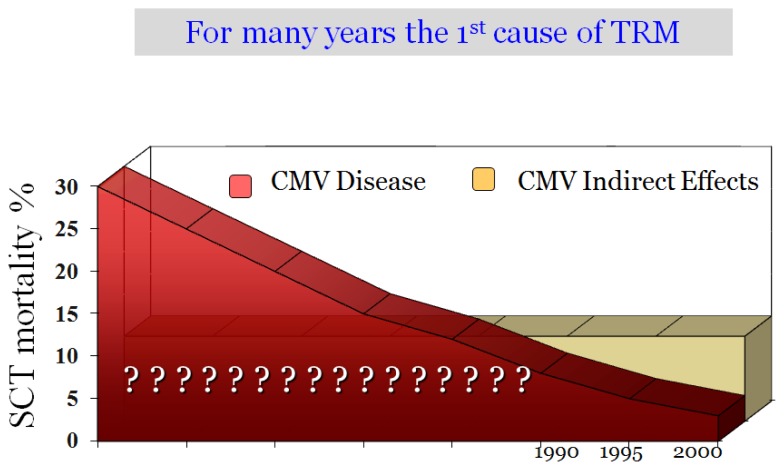


Mortality due to CMV-disease has decreased dramatically over time. In the 70’ and 80’, one every 5 patients died due to CMV disease, in the majority of cases due to CMV pneumonitis (figure 1). Today, the figure is around <2%. The control of CMV in stem cell transplantation (SCT) is probably the single advance with the highest impact in transplant survival in the last 25 years. What were the causes/reasons for this improvement? Certainly, there have been the advances in CMV prevention based on the development of diagnostic methods, such as antigenemia and PCR (both developed at the same time, 1988), and the development of anti-CMV antivirals such as ganciclovir (1989). Both developments allow the use of preventive strategies starting in the nineties that changed the CMV mortality dramatically. Today the incidence of CMV disease is <5%, according to the latest randomized trials (Table 1),^4^–^7^ and large review series.^8^ However, in contrast to these big advances in prevention, there have been few advances in therapy in the last 15 or 20 years (see later).

Another important change over time has been the type and time of presentation of prevalent type CMV disease, clearly related to the strategies for CMV prevention. Since the introduction of the preemptive therapy, the incidence of the viremia, as expected, remains unchanged in SCT recipients but there has been a significant decline in the incidence of early onset CMV disease (within first 100 days). Classically CMV pneumonitis was the main disease in SCT patients. The typical median time of presentation was between 50 to 60 days after transplant. It had a high attributed mortality (≥70%) and was the cause of the majority of CMV-deaths. Nonetheless, now, the gastrointestinal (GI) disease is the most frequent CMV disease in SCT (70–80%),^4^,^9^,^10^ because we are more efficient in preventing CMV pneumonitis than the CMV-gastrointestinal disease (GI). As a consequence the mortality of early CMV disease (within first 100 days) has decreased.^9^ The change to GI CMV disease, as the predominant form of CMV disease, seems to be related to the utilization of the PCR screening method instead of antigenemia^9^,^11^ or cultures,^12^ but not related to the change of the source of cells from bone marrow to blood or umbilical cord blood.^13^

It has been shown in several studies that, in contrast to CMV pneumonitis, antigenemia, and, to a lesser extent, also PCR have a low sensitivity for the diagnosis of GI CMV disease, ranging between 20–50%.^9^,^14^,^15^ These data suggest that CMV viral load in plasma does not adequately represent CMV replication in the GI mucosa probably because, in a proportion of patients, GI CMV disease represents a local event at least initially, in many cases associated to GVHD.

A consequence of the widespread use of preemptive therapy has been a switch from early to late CMV disease, so now late CMV disease, the disease that develops after day 100 from transplant, has become the predominant form of presentation in many transplant centers. Moreover, this is not a good thing. The proportion of CMV pneumonitis is higher in late disease (>50%),^16^ and these pneumonitides have the same high mortality as the early cases.

Another area of interest in the epidemiology of CMV in SCT is the possible impact that the new drugs might have. Some new drugs that a priori were not considered a risk for CMV have been later associated with the development of opportunistic infections including CMV disease in non-transplant patients, like ruxolitinib^17^,^18^ and Idelalisib.^19^ Patients receiving these agents prior to the transplant might have an increased risk of CMV, an issue that should be investigated. Moreover ruxolitinib is being used successfully for the treatment of refractory GVHD in an SCT patients setting of high-risk CMV infection and disease.^20^

#### a2) CMV definitions

We need widely accepted definitions of what are CMV infection and CMV disease in order to evaluate the impact of CMV in SCT across different studies and different centers. The current international definitions in use for CMV infection and disease were published nearly fifteen years ago.^21^ They claim that in the setting of SCT the term “CMV syndrome” should be avoided.

A central aspect of the definitions is the use of valid diagnostic tools. PCR alone is only sufficient for the diagnosis of a central nervous system disease, and not for other types of diseases like pneumonia or GI CMV disease. Detection of CMV by PCR alone may be too sensitive for the diagnosis of CMV disease and is therefore insufficient for this purpose. We have no data on what level of CMV DNA in bronchoalveolar lavage fluid or tissue correlates best with CMV disease, and therefore PCR is not recommended to make the diagnosis of CMV disease.^22^ In a small series the use of CMV-PCR in bronchoalveolar lavage was not clinically useful.^23^ Nonetheless, CMV PCR can be used in the diagnostic approach of a SCT patient with pneumonitis using a negative result to rule out CMV due to its high sensitivity and high negative predictive value.^22^,^24^

The problem now is that many centers do not have CMV culture available and depend on PCR for viral diagnosis. As a consequence in many SCT centers it is not possible to obtain a valid diagnosis of CMV pneumonitis without a lung biopsy, and even in this case the detection of CMV only by PCR will be considered not acceptable as definitive proof of CMV pneumonia or gastrointestinal disease. So in conclusion, we need new CMV disease definitions for a world based on PCR tools with no cultures.

#### a3) CMV management strategies

There are three strategies for the management of CMV: Prophylaxis, pre-emptive therapy and treatment of established CMV disease. The prophylaxis strategy is aimed at preventing all infections. The pre-emptive strategy consists in treating patients with high-risk infections to prevent disease. Moreover, finally when CMV disease is present, the aim of the treatment is to avoid organ damage and death.

##### Prevention of CMV complications

CMV prevention started in the eighties with the administration of CMV seronegative blood products^25^ and after, in 1995, with filtered blood products.^26^ The pre-emptive strategy era started in 1991^27^,^28^ and prophylactic ganciclovir strategy in 1993.^29^,^30^

As usual, big advances are made based on landmark studies with quite a few patients. In the case of preemptive therapy for CMV the proof of concept was established by the City of Hope-Stanford-Syntex CMV Study Group in an open, randomized study of 104 allogeneic SCT patients.^27^ Asymptomatic patients underwent bronchoalveolar (BAL) on day +35 post-transplant. CMV was evaluated in BAL by classic virologic techniques: shell-vial cell and conventional cell cultures, and cytology. Patients found positive for CMV (40 patients) were randomized to receive (20 patients) or not receive (20 patients) intravenous ganciclovir. At day +120 post-transplant, 75% of patients with positive CMV on BAL not treated developed CMV pneumonitis compared to 25% of those treated with ganciclovir, and 20% in those who were negative for CMV on BAL. This study proved the value of preemptive therapy for the prevention of CMV pneumonitis and started the era of the preemptive therapy for CMV. It is probably also the single study that has saved more lives in allogeneic SCT. A curious fact about this breakthrough study was the terminology used for the new strategy employed. The authors called their approach a “*Trial of prophylactic ganciclovir for cytomegalovirus pulmonary infection”,* apparently nothing new, no mention of the word “preemptive”. It was Robert. H. Rubin, in an editorial in the same number of the journal,^31^ who recognized the novelty of the new approach, different from prophylaxis and therapeutic approach coining the term “preemptive” therapy.

Although screening bronchoscopy was historically the first sample used to guide preemptive therapy, it was abandoned many years ago due to the clear superiority in efficacy and safety of the much more convenient sequential blood screening. Moreover, in a randomized trial, preemptive therapy based on antigenemia proved to be superior to preemptive therapy based on a day 35 screening bronchoscopy.^32^ CMV cultures were also abandoned in favour of non-culture techniques like antigenemia and PCR. In a randomized trial done more than 20 years ago^12^ PCR proved to be better than culture: PCR was associated with a lower rate of CMV disease and CMV-associated mortality, shorter duration of ganciclovir therapy, lower incidence and duration of severe neutropenia, and increased overall survival.

A randomized trial comparing prophylactic intravenous ganciclovir until day 100 post-transplant versus the preemptive ganciclovir therapy showed no significant difference in CMV disease by day 180 after transplantation and afterward (16.1% vs. 20.2%), and a similar overall survival. Nonetheless, prophylactic ganciclovir was associated with higher incidence of bacterial and fungal infections and increased use of ganciclovir. Thus, the preemptive use of ganciclovir guided by monitoring CMV viremia measured by antigenemia or qPCR became the standard of care in this setting.

##### Treatment of CMV disease

The treatment of CMV disease was based on noncomparative studies perform in the late eighties, establishing ganciclovir plus immunoglobulin as the treatment of choice for CMV disease, that was mainly pneumonitis at the time.^33^–^37^ Nonetheless, even with this treatment the mortality remain high (70%). The previous experience with monotherapy with ganciclovir, foscarnet or immunoglobulin did not improve the clinical outcome of CMV pneumonitis,^38^–^42^ and reviewed in.^43^,^44^

There is only one randomized trial that has compared ganciclovir with placebo in the treatment of CMV disease in allogeneic SCT patients,^45^ and it had disappointing results. A 14-day treatment course did not appear to influence clinical symptoms, the healing of gastrointestinal epithelium, the subsequent development of cytomegalovirus pneumonia, or overall mortality when compared with placebo.

In the meantime, has the treatment improved over time? Unfortunately, according to an extensive study, the outcome of CMV pneumonia showed only a modest improvement over time.^46^ In this study,^46^ with 421 CMV pneumonitis, the overall survival at 6 months was 30%, similar to the historical series. Outcome improved after the year 2000 showing a significant decrease in attributable mortality (adjusted hazard ratio, aHR, 0.6, P = 0.01), and a trend to a lower all-cause mortality: (aHR, 0.7, P = 0.06). Nonetheless, the effect of time may be due to changes in the prevalence of important risk factors over time, like mechanical ventilation, lymphopenia and hyperbilirubinemia.^46^

Moreover, what happens if the patient does well and survives CMV disease? Unfortunately, several studies have shown a grim outcome, mainly because a previous CMV disease is an independent risk factor for invasive aspergillosis (Hazard ratio 7.0)^47^,^48^ and candidemia (relative risk 16.4).^49^ In fact in these studies CMV disease was the-highest risk factor associated with IFI, greater than severe GVHD or the use of high dose of corticosteroids. Moreover, invasive aspergillosis was the most frequent cause of death in patients that survive a CMV disease. Based on these results, antifungal prophylaxis seems necessary for patients that survive CMV disease.

##### Cost

Another interesting aspect of the management of CMV today is the cost associated with the preemptive therapy of CMV infection. This evaluation has interest since now we can use new strategies to prevent CMV reactivation such as vaccines and adoptive immunotherapy that also have a high cost. A recent study compared the outcomes and post-transplantation treatment cost in 44 patients who never required pre-emptive CMV treatment with 90 treated patients. The treated group incurred an extra charge of $58,000 to $74,000 per patient.^50^

#### a4) Indirect effects

As previously mentioned, since the introduction of preemptive therapy a significant decline in the incidence of CMV disease has become the norm, now <5%, with a low CMV disease-related mortality (<2%). Nonetheless, CMV continues to be one of the leading causes of morbidity and mortality due to the so-called “indirect effects”. It was not until around 1990, that these indirect effects could be identified. Previously, the high CMV disease mortality precluded the detection of these effects. As shown in figure 1, these days SCT patients die more due to the indirect effects of CMV than due to CMV pneumonitis. CMV is associated with morbidity-mortality in 3 ways: CMV disease, the development of CMV infection, and by the presence of a positive serology pre-transplant.

It is recognized now that CMV causes mortality in 2 distinct ways: by the direct effects of the virus, in the form of a recognized viral disease, for example CMV pneumonitis; and by the so-called “indirect effects”, which are increasingly recognized as an important part of the whole viral effect. These indirect effects consist of clinical events associated with virus seropositivity or the development of viral infection, but not with the viral disease itself. These effects have been shown, not only in SCT patients but also in recipients of SOT and HIV patients. Several viruses have been described (different respiratory viruses, Herpesvirus type 6) but the paradigm of these direct-indirect viral effects is CMV. For CMV, the indirect effects outlined in the literature include: increased incidence of acute and extensive chronic graft-versus-host disease (GVHD),^51^,^52^ increased risk and mortality due to bacterial and fungal infections,^47^,^53^–^55^ and what is more important an increase in transplant-related mortality and a decrease in overall survival. In table 2 there is a summary of studies showing the negative impact of CMV seropositivity on the outcome of HSCT, in more than twenty-nine thousand patients. These adverse effects have been described mainly in patients who received depleted transplant or transplants from unrelated donors. However, they also occur in HLA-identical siblings transplants^51^,^56^ as showed in a large EBMT retrospective study in more than 56.000 patients, 72% of them from HLA-identical siblings:^57^ CMV seropositive patients had a higher mortality compared to transplant where both patient and donor were CMV seronegative.

A recent CIBMTR analysis on 9,469 patients transplanted between 2003 and 2010,^58^ showed that CMV reactivation was associated with inferior OS among all disease groups in multivariate analysis confirming that today, in spite of widespread preemptive therapy, CMV reactivation continues to remain a risk factor for poor post-transplant outcomes.

#### a5) Trends in Allogeneic Transplantation: an increase of patients at risk of CMV complications

Changes in the age and origin of the patients and the increasing use of unrelated donors from countries with a very low HCMV prevalence are causing an increasing proportion of transplants in CMV-seropositive patients from CMV-seronegative donors, a combination that has been associated with a worse outcome than a +/+ combination and of course −/− pairs of receptor-donors.^59^ This is due to 2 facts. First, there are an increasing proportion of seropositive patients due to the clear increase in their age, and second, there is a decrease in the percentage of CMV seronegative donors.^60^

It is well known that CMV seroprevalence has a strong correlation with age. In the US population, for example, 54% of patients younger than 40 are CMV seropositive compared to 83% in those of ≥60 years.^61^ One of the most significant tendencies in allogeneic SCT is the increase in the age of the recipients. During the decade 2002–2011, patients older than 60 years doubled from 8% to 17%, increasing to 22% of allogeneic transplant recipients in 2007–2013.^62^ This implies that more allogeneic transplant patients are CMV seropositive. Moreover, allogeneic SCT are increasing in parts of the world that previously had a low rate of activity. This is the case of Latin America, where the seroprevalence of the population is higher than in Europe of North America. This translates into greater resources for the management of CMV for Latin American transplant centers.^63^

#### a6) CMV: a troll or a warrior of transplantation?

As previously mentioned, CMV has been called the troll of transplantation mainly due to the high mortality associated with CMV pneumonia. Nonetheless, CMV has also been associated, almost three decades ago, with a decrease in leukemic relapses after SCT, particularly in acute myeloid leukemia and chronic myeloid leukemia (CML), but to a lesser extent in myelodysplastic syndrome, acute lymphoblastic leukemia and Non-Hodgkin lymphoma patients. It was first reported by Lönnqvist et al, in a small cohort study, that CMV infections were associated with a decrease in relapses in leukemic patients.^64^ More recently an impressive study by Elmaagacli et al reactivated the interest on the role of CMV in decreasing relapses after SCT, defining this association as “virus-versus-leukemia” effect.^65^ In this study early CMV replication was associated, in the univariate and multivariate analysis, with a marked decrease in relapse (at 10 years: 9% vs. 42%, P 0.0001) and with an increase in survival (at 10 years: 62% vs. 37%, P 0.005). Based on these results CMV can also be seen as a warrior of transplantation. Nonetheless the relation between CMV infection and leukemia recurrence in patients with hematologic malignancies after allogeneic SCT has been a highly controversial issue for many years. Contradictory results have been obtained in different studies, based on CMV serology, CMV infection, and even with both techniques. There are more than 30 studies that have evaluated the role of CMV in relapse, but their detailed analyses are outside the scope of this review. It can be said that usually multicentric studies do not find a protective effect of CMV on relapse, being unicentric studies those who find it. In the 5 largest studies, with more than 96,000 patients, no effect of CMV (serology or infection) on relapse was found.^57^–^59^,^66^,^67^ Nonetheless, it is an interesting aspect that requires more studies.

### b) Management Today

#### b1) Guidelines for CMV management in SCT

There are several guidelines for CMV management although the two most widely accepted are the 2008 European Conference on Infections in Leukemia (ECIL) guidelines^68^ and the 2009 international consensus guidelines,^69^ both quite similar.

##### Prevention of CMV complications

What do we want to prevent: infection or CMV disease? This is an important question as it has an impact on the strategy we select. A preemptive strategy can only decrease CMV disease incidence, but prophylaxis may have an effect on the complications produced by CMV infection and even on those associated with CMV positive serology. Until now, for SCT patients and with the available antivirals, the aim has been to prevent CMV disease, and the strategy of choice for the majority of the patients is preemptive therapy.^68^,^69^

A summary of the diagnosis and prevention recommendations appears in Table 3. Ganciclovir is often used as a first-line drug for preemptive therapy. Although foscarnet showed in a randomized trial to be as effective as ganciclovir and with a lower incidence of severe neutropenia,^70^ it is currently more commonly used as a second-line drug, because of practical reasons.

Those patients that have only one reactivation episode after SCT are usually treated successfully with the available antivirals. Problematic patients are those that suffer repetitive replicative episodes, typically in the setting of GVHD treated with intensive immunosuppression, or transplants done with profound T cell impaired immune reconstitution, due to T-cell depletion (in vivo or in vitro) or a low T-cell dose on the graft (cords). These patients are prone to devastating CMV complications, like CMV encephalitis, a form of CMV disease with >90% mortality today.^71^

For patients with profound T cell impaired immune reconstitution the use of cell adoptive immunotherapy is being applied with increasing success. For patients with intensive immunosuppression, particularly high dose of corticosteroid, adoptive cell therapy needs further technical refinements that are still experimental.^72^ These technologies seem not to be associated with significant toxicity but their effectiveness needs to be further assessed in controlled trials^68^ and cannot be recommended.^69^ Nonetheless, this interesting approach is not reviewed here in more detail. There are several good recent updates for the interested reader.^72^,^73^

##### Treatment of CMV disease

The standard treatment for CMV pneumonia, based on small studies in the late 80’ is the combination of intravenous ganciclovir plus immune globulin as recommended by the ECIL group.^68^ For other types of CMV disease monotherapy with ganciclovir or foscarnet with immune globulin is recommended (Table 4).

Although recommended in the guidelines, do we need combined therapy with immune globulin for CMV pneumonitis? Based on a recent publication^46^ the answer is probably no. In this large retrospective study on 421 CMV pneumonia episodes in SCT patients the use of combined therapy (ganciclovir or foscarnet) with immunoglobulins, compared with antiviral monotherapy did not show an impact on CMV pneumonia mortality (global or CMV-related). Contrary to early studies of ganciclovir as monotherapy this study is the best evidence to date that monotherapy with ganciclovir or foscarnet had a beneficial effect vs. no therapy. The effect of antiviral monotherapy on overall survival compared with no treatment was significant in both univariate and adjusted models. Another smaller study did not see an impact on the association of intravenous immune globulin with the response of CMV pneumonitis.^74^ As the role of immune globulin for the treatment of CMV is questionable, if it is used, an unspecific immunoglobulin is recommended over the CMV-specific, that is much more expensive and harder to obtain.

So inclusion, even today, it is better to prevent than to treat CMV disease, and combined therapy with intravenous immune globulin seems to be no better than monotherapy with antivirals (ganciclovir or foscarnet).

#### b2) Today antivirals

For the management of CMV we have both high and low potency antivirals (Table 5). Low potency antivirals can be used for prophylaxis but have no role in the treatment of infections or disease by CMV. For these CMV complications high potency antivirals are needed.

Two drugs have been key in antiviral development: acyclovir and ganciclovir. Acyclovir was discovered by Gertrude Elion and George Hitchings in 1977 at the Wellcome Research Laboratories, which was one of the discoveries that won them the Noble Prize in 1988. The discovery of acyclovir, the first efficient and safe drug for the treatment of herpes virus infections, opened a new era of antiviral therapy. Ganciclovir was discovered in 1980 by Dr. Kelvin Ogilvie and his research team at McGill University. Ganciclovir, the first high potency anti-CMV agent was first used in a human in 1984 to treat a bone marrow transplant patient. It was launched in 1989. Later came foscarnet, cidofovir and Valganciclovir.

The mechanism of action of all the classical anti-herpesvirus agents is the inhibition of viral DNA polymerase. All of these antivirals, except foscarnet, require phosphorylation by cellular kinases. Ganciclovir, acyclovir, penciclovir, and brivudine also require an initial phosphorylation by a virus-encoded kinase. In the case of ganciclovir the encoded protein kinase pUL97 of the virus performs the first phosphorylation. Foscarnet directly binds to the pyrophosphate binding site of the viral DNA polymerase without any intracellular chemical modification.

The efficacy and safety profile are two essential aspects of the antivirals for the management of CMV. The low potency antivirals, aciclovir and valaciclovir, have a good safety profile and can be given orally or by intravenous route (aciclovir). In contrast the available high potency antivirals for CMV (ganciclovir, foscarnet and cidofovir) have several and significant drawbacks:

- There is no approved oral agent for SCT. Valganciclovir, although widely used in the clinic has no phase III study in SCT patients and it is not registered for this population.
- The available anti-CMV agents have substantial toxicities, and this is one of the major reasons that make preemptive therapy the strategy of choice. The main toxicities of these drugs are nephrotoxicity, myelosuppression and delay immune reconstitution.^75^ Ganciclovir cause neutropenia in at least 20–30% of the cases, which is an independent negative risk factor for overall survival, disease-free survival and transplant related mortality.^76^ Therefore, ganciclovir/valganciclovir are not adequate for neutropenic patients, and in prophylaxis they have to start late after full engraftment. Moreover, the prolonged use of ganciclovir (more than 4 weeks) is a risk factor for the development of late CMV disease and invasive Aspergillosis.^77^ With each week of ganciclovir treatment the risk of invasive aspergillosis increases by a factor of 1.4.^78^ Ganciclovir produces a significant increase of aspergillosis that is independent of secondary neutropenia and proven CMV infection.^78^ Both ganciclovir/valganciclovir and foscarnet are nephrotoxic and require dose adjustment with renal impairment.
- The use of these drugs in the prophylaxis or as preemptive treatment do not prevent the indirect effects of CMV, with only one exception. In a retrospective study preemptive treatment significantly decreased the risk of extensive chronic GVHD.^79^

The different toxicities and activity of anti-CMV antivirals have different clinical implications according to the results of several prophylactic studies. High-dose aciclovir, a low potency but low toxic antiviral, compared to placebo, decreased CMV viremia by 40%, had no impact on CMV disease incidence, but was associated with a 20% increase in overall survival.^80^ Curiously, high dose of acyclovir was associated with a near 20% decrease in deaths due to infection (11% vs. 28%). A previous no randomized study showed that high-dose prophylactic acyclovir reduced CMV infection by 30%, and increased overall survival from 46% to 71%.^81^ In a retrospective study, the use of 1 year of acyclovir or valacyclovir prophylaxis was associated with improved overall survival in allogeneic SCT.^82^ In a later randomized study that compared high dose of valaciclovir with a high dose of aciclovir, valaciclovir proved to be more effective than acyclovir, decreasing by 40% the rate of CMV infection and the use of ganciclovir/foscarnet.^83^

In contrast, ganciclovir, a high potency and higher toxic antiviral, compared to placebo, in two randomised trials produced a greater decrease in CMV infection (70–90%), a decrease in early CMV disease, an increase in the incidence of severe neutropenia, but was not associated with survival increase.^29^,^30^ Although not statistically significantly more patients died of infections in the prophylactic ganciclovir group in both studies (50% vs. 25%; and 17% vs. 6%). Moreover, in a randomized trial, intravenous ganciclovir until day 100 after transplantation was no better than high-dose valacyclovir in the prevention of CMV infection (12% vs. 19%) or CMV disease (1/85 vs. 2/83).^84^ Nonetheless, the ganciclovir group had a significantly higher incidence of neutropenia (32% vs. 13%), and a non-significant increased incidence of infections after engraftment.

#### b3) CMV management in SCT: an “art” that is center-dependent

Preemptive antiviral therapy has been adopted by most centers as the strategy of choice for the prevention of CMV disease in the allogeneic STC. However, it is a fact that the strategies apply at the centers differ notably. Although quantitative real-time PCR assays (qRT-PCRs) have largely replaced the pp65 antigenemia assay for the guidance of preemptive antiviral therapy, many differences exist among centers. There are variations in the type of sample used (plasma vs. whole blood), in the method of DNA extraction (manual vs. automated techniques), type of technique (commercial vs. homemade), and in the quantitative viral load interpretation, the latter being one of the most important factors. Some centers use the same value of PCR for all patients and others that use different PCR cut-offs for different risk populations.^22^ Moreover, finally, the threshold used to start preemptive therapy shows an enormous variation from center to center (100, 200, 500, 600, 1000, 10,000, 50,000 copies/ml, to put some examples).^22^,^85^–^90^

A recent national survey revealed striking differences among centers in CMV surveillance practices for the prevention of CMV disease, especially regarding the criteria that triggered the start of preemptive antiviral therapy, yet the overall incidence of CMV end-organ disease was reported to be <3%, with minor variations among centers.^91^ In this study, all centers used a no-risk adapted preemptive strategy, and all used a qRT-PCR with fully automated DNA extraction (except 1 center). The threshold for start therapy showed considerable variation among centers: 100 – 5000 copies/ml for plasma, and 400 – 1000 copies/ml for whole blood. Curiously, 9 centers using the same commercial technique and the same DNA extraction method showed variation in the threshold to start preemptive therapy (150–5000 copies/ml).

One of the problems with the different qRT-PCRs used was that they were not directly comparable because of intrinsic differences in the performance of the assays and the nature of the calibrator. The same sample with an expected result of 1000 copies/ml studied at different centers gave different results that vary from 0 to 20,000 copies/ml.^92^ In 2010 the first international standard for CMV QNAT was established by the World Health Organization (WHO) Expert Committee on Biological Standardization.^93^ PCR viral loads are expressed in international units/ml. Will the use of normalized values to IU/ml decrease these variations? The answer is no, at least at a significant level. For example in the above mentioned national survey commented,^91^ the above-described differences even stood out when CMV DNA loads (in copies/ml) were normalized to IU/ml, according to the conversion factor recommended by the respective manufacturer.

So, we have to conclude that the preemptive strategies employed at different centers are probably unique to each center, and CMV management in SCT is therefore an “art” that is center-dependent.

### c) What is coming?

#### c1) New antivirals

In spite of the successful prevention of early CMV disease with the present preemptive or prophylactic strategies, CMV still causes important morbidity and mortality. Until now, due to the toxic effects associated with the anti-CMV drugs available, the use of prophylaxis has not been associated with improved outcome. In contrast the use of low prophylactic potency but low toxic antivirals (acyclovir, valacyclovir) was associated with an increase in survival in one randomized study^80^ and 2 controlled studies.^81^,^82^ The development of new potent antivirals with an excellent safety profile is an unmet need in SCT. These and other evidence strongly support the idea that a high potency but low toxic anti-CMV drug has a good rationale for improving the outcome of SCT patients.

Anti-CMV agents have been difficult to develop. The last new compound was approved in 1995 (cidofovir) and the latest new formulation in 2001 (valganciclovir). Quite unexpectedly, three new antivirals have been developed recently more or less at the same time. As Prof. Griffiths graphically express in an excellent editorial comparing new antivirals and commuters in London: “you wait ages for a bus and then three come along at the same time”.^94^ The same has happened for new anti-CMV agents. These three new antivirals are: maribavir,^95^ brincidofovir^96^ and letermovir (Table 6).^97^

##### Summary of the new anti-CMV antivirals

None is approved for any indication and none of them are nephrotoxic or myelotoxic.

The antiviral spectrum is quite different (Table 6). Letermovir is a pure anti-CMV agent; maribavir shows activity against CMV and EBV; and brincidofovir has the broadest spectrum of all anti-DNA viruses, broader than cidofovir.

All have a convenient posology: maribavir once every 12h; letermovir once a day; and brincidofovir twice weekly. Maribavir and letermovir have an entirely different mechanism of action compared with the traditional anti-herpes agents.

Maribavir binds to UL97 and inhibits the assembly, encapsidation and nuclear egress. Letermovir inhibits CMV terminase complex. (UL56), but not DNA-polymerase. Moreover, brincidofovir, a lipid conjugate of cidofovir, inhibits viral DNA-polymerase as does cidofovir.

All showed positive results in prophylactic phase II trials in allogeneic SCT(table 6).^5^,^6^,^98^ They were more effective than placebo and showed a good safety profile. Maribavir is practically atoxic with taste disturbance as the most frequent adverse event. Brincidofovir causes diarrhea with dose-limiting toxicity at a dose of 200 mg twice a week or higher.

However, unfortunately, and quite unexpectedly, two of them, maribavir^4^ and brincidofovir,^99^ failed primary efficacy end-point in prophylactic phase III trials in SCT patients. Several reasons have been given for the failure of maribavir, with the low suboptimal dose being one of the main causes (100 mg bid).^100^ A note of caution has to be made about the phase III trial of brincidofovir because only a preliminary presentation is available^99^ and not the final publication. Nonetheless, it appears that one of the major drivers of the failure might be the diagnosis and management of brincidofovir induced diarrhea as GI-GVHD in many cases. The phase III trial of prophylactic letermovir in allogeneic SCT is ongoing and no results are available.

The clinical use of maribavir at high doses has shown a 50–66% response rate in several refractory or resistant cytomegalovirus infections the majority with CMV disease.^101^,^102^ These are encouraging results that merit further study. Maribavir has completed 2 Phase II randomized studies of treatment of CMV infection with high doses (400-800-1200 mg BID), as first-line treatment (EudraCT Number: 2010-024247-32) or as salvage therapy for CMV infections that are resistant or refractory to ganciclovir/valganciclovir or foscarnet (NCT01611974). These doses are higher than those employed in the phase III trial (100 mg bid).^4^

Due to its broad spectrum of activity, brincidofovir is being studied in other non-CMV infections. Of these, adenovirus is the infection in the most advanced phase of development with a phase III trial running now.^103^

We have to admit that the development of a successful anti-CMV drug is becoming harder than expected. We now have more questions than answers: Would they finally be effective in prophylaxis, preemptive or directed therapy for CMV? Would they change the paradigm from preemptive to prophylactic strategy? What impact will they have on relapse? Will they have an impact on the indirect effects? Hopefully, one or more of these new three antivirals will be approved for use in SCT patients. As previously mentioned the development of new antivirals with high anti-CMV potency, and a good safety profile is an unmet need in SCT.

The knowledge of the biology of CMV could bring new routes for the development of new drugs or a new use of old ones. It has recently been reported that the inhibition of mitochondrial translation with chloramphenicol abolished the HCMV-mediated increase in mitochondrially-encoded proteins and significantly impaired viral growth.^104^

#### c2) CMV vaccine

The prevention of CMV complications by CMV vaccine has a long unsuccessful history.^105^ In allogeneic SCT patients, in a randomized, double-blind, placebo-controlled trial, a plasmid DNA vaccine that contains two plasmids that encode gB y pp65, showed to be effective (50% decrease in viremia), well tolerated and able to induce serologic and specific T cell responses against CMV.^106^ Based on this good result the vaccine is now undergoing a Phase III trial for CMV prophylaxis in allogeneic SCT patients.

### Unmet Medical Needs Regarding CMV in SCT Recipients

To end this review I would like to point out what are, in my opinion, the unmet medical needs regarding CMV in SCT recipients:

- New CMV disease definitions for the world based on PCRs tools with no cultures.
-The development of potent, non-toxic anti-CMV drugs, particularly with no toxicity to the kidneys, haematopoiesis and immunologic recovery.
-A better preemptive therapy with consistent thresholds across centers for the beginning and end of preemptive therapy.
-Improvement in the treatment of CMV disease, a field without clear progress in the last 25 years.
-An effective strategy for prevention of late CMV disease.
-And last, but not least, the reduction of the impact of the indirect effects in CMV seropositive patients.

## Figures and Tables

**Table 1:** CMV Disease incidence in the preemptive era. Incidence of CMV disease in the placebo groups in randomized trials.

Drug	Study	N∘ Patients	CMV disease incidence
**Maribavir**	Marty FM, Lancet ID 2011 (4)	**227**	**2.4% (at 6 months)**
**Brincidofovir**	Marty FM. NEJM 2013 (5)	**59**	**3.0% (at 3 months)**
**Letermovir**	Chemaly RF. NEJM 2014 (6)	**33**	**0% (at 3 months)**
**Valganciclovir**	Boeckh M, Ann I. Med. 2015 (7)	**89**	**2.0% (at 9 months)**

**Table 2:** CMV Indirect effects: impact of + serology (from table 1, Boeckh M. Blood 2004;103:2003 with modifications^*^).

Author (year)	Patient N∘	TCD %	UD %	Results (P <0.01) R+ vs R/D CMV (−)
Broers (2000)	115	**95**	0	**24%** ⇓ **absolute OS**
Cornelissen (2001)	127	26	**100**	**38%** ⇓ **relative DFS**
Craddock (2001)	106	**100**	**100**	**22%** ⇓ **absolute OS**
Doney (2003)	182	0	52	**99%** ⇑ **relative TRM**
Kollman (2001)	6978	25	**100**	**7%** ⇓ **absolute OS**
Kroger (2001)	125	**100**	**100**	**41%** ⇓ **absolute OS**
Ljungman^*^ (2014) (59)	8801**	**100**	**69**	**5%** ⇓ **absolute OS**
MacGlave (2000)	1423	23	**100**	**20%** ⇓ **relative DFS**
Malaspina (2002)	510	24	**100**	**46%** ⇓ **relative DFS**
Meijer (2002)	48	**100**	**100**	**41%** ⇑ **absolute TRM**
Nichols (2002)	1750	0	57	**26%** ⇓ **relative OS**
Teira^*^ (2016) (58)	9469	52	29	**60** ⇑ **relative TRM**
Yakoub-Agha^*^ (2006) (107)	236	0	23	**16%** ⇓ **absolute OS** **14%** ⇑ **absolute TRM**

TCD: T-cell depletion. UD: unrelated donor.

** This is a subpopulation of the study, restricted to the impact of using a CMV Seropositive Donor for a CMV-Seronegative unrelated patient.

**Table 3:** Guidelines for CMV management in SCT: Prevention of CMV disease in allogeneic-SCT. ECIL recommendations^68^

**Diagnosis** The diagnosis of CMV disease must be based on symptoms and signs consistent with CMV disease together with detection of CMV by an appropriate method applied to a specimen from the involved tissue (A II) ○ Symptoms of organ involvement + CMV detection in blood are not enough for diagnosis of CMV disease PCR is usually not appropriate for documentation of CMV disease in tissue specimens, as the PPV is too low (B III)
**Monitoring** All allogeneic-SCT patients, regardless of whether they receive CMV prophylaxis, should be monitored for CMV in peripheral blood at least weekly using either CMV antigenemia assay or a technique for the detection of either CMV DNA or RNA (AI). Use of a quantitative assay gives additional information valuable for patient management (B II). The duration of monitoring should be at least 100 days (BIII). Longer monitoring is recommended in patients with acute or chronic GVHD, in those having experienced CMV infection after SCT earlier and in those having undergone mismatched or unrelated donor transplantation (BII).
**Prevention** The strategy of choice: pre-emptive therapy ○ Pre-emptive antiviral therapy based on detection of CMV antigen or nucleic acid (A I) ○ Either intravenous ganciclovir or foscarnet can be used for first line pre-emptive therapy (A I) ○ Valganciclovir might be used in place of i.v. agents especially in low-risk patients (provisional BII). ○ Cidofovir can be considered for second-line pre-emptive therapy (3–5 mg/kg) but careful monitoring of renal function is required (BII). Prophylaxis ○ Iv ganciclovir prophylaxis could be used in sub-groups of patients at high risk for CMV disease (BI) (not specified). ○ Acyclovir or valacyclovir can be used as prophylaxis against CMV in allo-SCT patients (BI). However, their use must be combined with monitoring and the use of pre-emptive therapy (AI). ○ Immune globulin has no role as prophylaxis against CMV infection (EII). Adoptive cellular immunotherapy ○ Infusion of CMV specific lymphocytes or Dendritic cell vaccination are interesting options and should undergo controlled prospective clinical trials (C II)

**Table 4:** Guidelines for CMV management in SCT: CMV disease treatment. ECIL recommendations^68^

CMV pneumonia (allo-SCT) ○ Ganciclovir is recommended (AII) ○ Foscarnet might be used in place of ganciclovir (AIII) ○ The addition of immune globulin to antiviral therapy should be considered (CII) ○ Cidofovir or the combination of foscarnet and ganciclovir can be used as second-line therapy (BII). Other types of CMV disease and in other patients groups ○ Ganciclovir or foscarnet without Ig is recommended (BII) ○ Cidofovir or the combination of i.v. ganciclovir and foscarnet can be used as second-line therapy for CMV disease (BII).

**Table 5:** Present CMV antivirals

Drug	Route	Approval
High potency		
• Ganciclovir	(iv)	1989
• Foscarnet	(iv)	1991 Aids
• Cidofovir	(iv)	1996 Aids
• Valganciclovir (not registered for SCT)	(oral)	2001 Aids 2003 SOT
• Fomivirsen	(intravitreal)	1998*
Low potency		
• Acyclovir	(oral, iv)	1982
• Valacyclovir	(oral)	1995

* Was voluntarily withdrawn from the European market in 2002

**Table 6:** New anti-CMV antivirals

Drug	Mechanism of action	Route	Spectrum	Prophylactic studies in allogeneic SCT	
				Phase II	Phase III

**Maribavir**	UL 97 inhibition	ORAL	CMV and EBV	Winston DJ, 2008 (98): 111 patients Primary end-point: Success Failures: 7% vs. 46% placebo	Marty FM, 2011 (4): 681 patients Primary end-point: Failure

**Brincidofovir**	Viral DNA polymerase inhibition (UL 54)	Oral	The broadest (CMV, EBV, adenovirus,…)	Marty FM, 2013 (5): 230 patients Primary end-point: Success Failures: 10% vs. 37% placebo	Marty FM, 2016 (99): 458 patients Primary-end-point: Failure

**Letermovir**	CMV terminase complex inhibition (UL 56)	Oral & iv	CMV	Chemaly RF, 2014 (6): 132 patients Primary end-point: Success Failures: 29% vs. 64% placebo	Ongoing
